# Efficacy of intraoperatory optimisation of fluids guided with transoesophageal Doppler monitorisation: a multicentre randomised controlled trial

**DOI:** 10.1186/cc12143

**Published:** 2013-03-19

**Authors:** S Maeso, R Villalba, J Ripollés, S Asuero, J Blasco, J Calvo

**Affiliations:** 1 Agencia Lain Entralgo, Madrid, Spain; 2 Hospital Universitario Ramón y Cajal, Madrid, Spain; 3 Hospital Infanta Leonor, Madrid, Spain

## Introduction

The objective is to compare stay in surgery monitored with oesophageal Doppler with unmonitored.

## Methods

A randomized trial. We present preliminary results obtained in the first 55 cases. The surgeries were general and urological. ISRCTN93543537.

## Results

There were no differences in any of the baseline variables. A total of 69.1% were men. The mean age was 65.80 years. There were 63.6% general surgery and 36.4% urologic surgery. There were 81.5% open surgeries and 18.5% laparoscopic. The results were favorable to the intervention group for most outcomes; these differences did not reach statistical significance (Table 1). We emphasize the difference in postoperative stay of 3.4 days. Only complications results were against the intervention.

**Table 1:** Analysis of the outcome variables

Outcome	Units	ODM (*n *= 32)	Control (*n *= 23)	*P *value
Surgery time	Minutes	206.20	225.59	0.532
Total stay	Days	13.92	18.19	0.292
Postsurgery	Days	10.93	14.36	0.280
Time to enteral	Days	2.73	3.13	0.567
Time to deambulation	Days	2.52	3.00	0.359
Complications	%	34.4	21.7	0.377

## Conclusion

The preliminary results obtained for the postsurgical length of stay, as for most of the outcomes, were favorable to monitoring by oesophageal Doppler.

**Figure 1:**
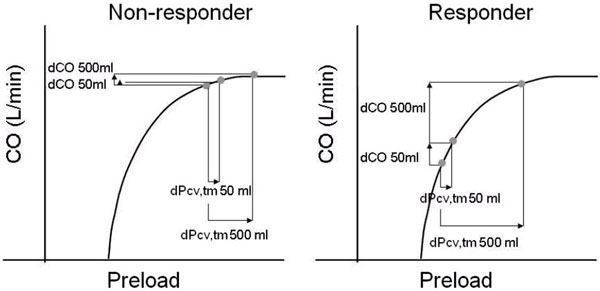
**Cardiac function curve: a fluid challenge of 50 ml in a (non)responder**.

